# Challenges and opportunities of integrating imaging and mathematical modelling to interrogate biological processes

**DOI:** 10.1016/j.biocel.2022.106195

**Published:** 2022-05

**Authors:** Maxime Berg, Natalie Holroyd, Claire Walsh, Hannah West, Simon Walker-Samuel, Rebecca Shipley

**Affiliations:** a UCL Mechanical Engineering, Torrington Place, London WC1E 7JE, UK; b UCL Centre for Advanced Biomedical Imaging, Paul O’Gorman Building, 72 Huntley Street, London WC1E 6DD, UK

**Keywords:** Biophysical modelling, Validation, Multiscale

## Abstract

Advances in biological imaging have accelerated our understanding of human physiology in both health and disease. As these advances have developed, the opportunities gained by integrating with cutting-edge mathematical models have become apparent yet remain challenging. Combined imaging-modelling approaches provide unprecedented opportunity to correlate data on tissue architecture and function, across length and time scales, to better understand the mechanisms that underpin fundamental biology and also to inform clinical decisions. Here we discuss the opportunities and challenges of such approaches, providing literature examples across a range of organ systems. Given the breadth of the field we focus on the intersection of continuum modelling and in vivo imaging applied to the vasculature and blood flow, though our rationale and conclusions extend widely. We propose three key research pillars (image acquisition, image processing, mathematical modelling) and present their respective advances as well as future opportunity via better integration. Multidisciplinary efforts that develop imaging and modelling tools concurrently, and share them open-source with the research community, provide exciting opportunity for advancing these fields.

## 1 Introduction

Recent breakthroughs in imaging (Abdeladim et al., 2019, Chakraborty et al., 2019), mathematical and computational sciences (Hartung et al., 2021) have created exciting opportunities to integrate these approaches and interrogate biological processes at an unprecedented level. Cutting-edge imaging technologies provide insight into both structure and function of tissues in health and disease, in terms of fundamental biology (Walsh et al., 2021) and clinical outcomes (Wen et al., 2019). Mathematical modelling provides a framework to integrate such data sets, test hypotheses and make both qualitative (Berg et al., 2020) and quantitative (Epp et al., 2020, Pearce et al., 2016) predictions that would be challenging using experimental assessments in isolation, and can guide diagnostic and therapeutic strategies (Coy et al., 2021).

Not only does the integration of imaging and mathematical approaches remain a significant research challenge, but it is also driving advances in both fields individually. Predicting the evolution of disease for individual patients, for example through a mathematical-imaging framework, is a holy grail in numerous diseases. This is inspiring the development of new techniques to image tissue microstructure and function in situ, at high resolution and through time, so that the spatio-temporal development of individual tissues can be better understood. Concurrently, pathology-specific mathematical models are being developed alongside computational tools to analyse the volume of imaging data being produced (d’Esposito et al., 2018, Gagnon et al., 2016). Mutual validation of such modelling predictions and spatio-temporal imaging data remains challenging yet is an essential feature of the research field moving forwards.

**Fig. 1:**
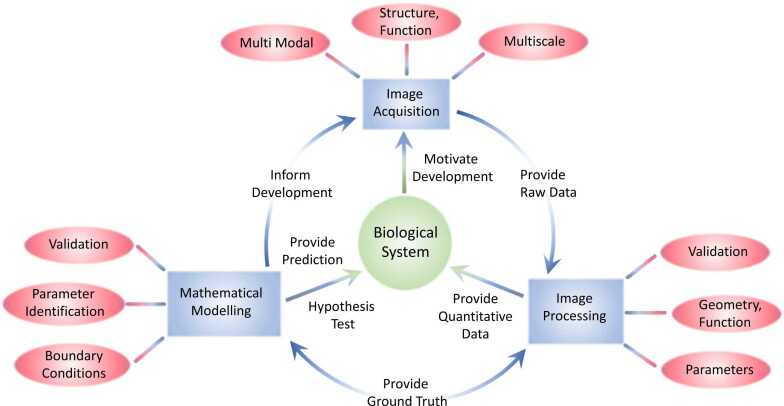
Synergetic relationships between imaging and mathematical modelling. Each rectangle represents a technology used to study a biological system (image acquisition, image processing, mathematical modelling). Each modality has an associated set of challenges (ellipses) that can be overcome by integrating all three disciplines to form an iterative cycle, motivated by the need to better understand complex biological systems.

We identify three pillars of research key to these goals (image acquisition, image processing and mathematical modelling) and discuss their opportunities and challenges relevant to interrogating biological processes (Fig. 1). We provide examples from blood circulation and perfusion, an established field in which an active community in both imaging and multiscale modelling interact across basic and applied science. However, there are numerous other areas (for example, transport phenomena, solid mechanics and cell dynamics), which are advancing on a similar trajectory and face analogous challenges and opportunities. Across all these areas, progress will be accelerated through open-source sharing of complete, fully annotated and user-tested model and data sets. Two cutting-edge exemplars are the Chaste (Cancer, Heart and Soft Tissue Environment) simulation package for multi-scale, computationally challenging problems in biology and physiology (https://www.cs.ox.ac.uk/chaste/) and the Human Organ Atlas of hierarchical phase-contrast tomography data from human organs (https://human-organ-atlas.esrf.eu).

## 2 Image acquisition

A range of imaging modalities are used to probe the structure and function of biological systems for example optical microscopy, ultrasound, magnetic resonance imaging (MRI) and X-rays. Modality choice depends on factors including resolution, field-of-view, depth penetration, source of contrast, in-vivo compatibility and dimensionality (2D vs 3D). The ultimate goal is to obtain entire 3D tissue structures, in-vivo*,* at high resolution.

Optical imaging provides high-resolution data with established labelling strategies, however poor penetration complicates in vivo application. Techniques including multi-photon microscopy – in which fluorophores are simultaneously excited by multiple photons, reducing background signal and increasing tissue penetration – have been used to create graphical models of cerebral blood vessel networks (Damseh et al., 2019). Optical techniques are increasingly being combined with tissue clearing, where chemical refractive index matching renders tissue transparent, thus increasing penetration but preventing in vivo use (d’Esposito et al., 2018).

MRI and photoacoustic imaging can provide functional in vivo measurements, for example blood flow. Diffusion-weighted MRI, in which the signal is mediated by the diffusion of water through biological tissue, has been combined with computational models to predict micro-structural features of brain tumours (d’Esposito et al., 2018, Johnston et al., 2019). Photo-acoustic imaging generates an ultrasound signal via laser-induced thermoelastic expansion of tissue. It has good depth penetration and is in vivo-compatible, but lacks the resolution of the other optical techniques (Daly and Leahy, 2013).

Finally X-rays can provide high resolution images with multiscale techniques bridging from the micron to the whole organ scale (Walsh et al., 2021); however, labelling strategies are underdeveloped and radiation dose can limit resolution in vivo.

Combining multiple imaging modalities to leverage the strengths of each can be an effective strategy. For example, (d’Esposito et al., 2018) combined OPT of cleared samples to provide accurate geometric representations of tumour blood vessel networks with MRI to quantify perfusion. The vessel geometries were used as substrates for computational models of blood flow, in turn validated through the MRI measurements.

Despite these significant advances, resolving complete tissue architectures at high resolution, in-vivo*,* remains unsolved. Further, as imaging becomes more sophisticated, datasets become larger (10 GB-1TB is now routine). Processing these large datasets and integrating them with computational modelling, remains a significant challenge (Bernabeu et al., 2020).

## 3 Image processing

Image processing aims to extract geometric objects and functional information from raw image data. Such data can provide substrates as well as validation options during mathematical model development (Fig. 1). However, the complexity and variability of image data as well as the lack of ground truth, mean that image processing is inherently challenging (Nguyen et al., 2020).

Segmentation is a crucial step - given a raw image, segmentation categorises image pixels into within or outside a target object. Segmented objects may be discretized into forms such as meshes, points, or spatial graphs.

Segmentation and discretisation methods are a vast field of research in their own right (Wang et al., 2020) and method selection and optimisation must consider variation between samples e.g. healthy versus pathological images, as well as the specific modelling requirements. Multiple image processing methods may be required to generate all required model inputs (d’Esposito et al., 2018). Analogously, adjustments in the imaging modality or sample preparation will likely necessitate re-optimisation or new image processing method development (Bernabeu et al., 2020, Cebulla et al., 2014).

Machine learning (ML) approaches to image segmentation, in particular Encode-decoder networks, have rapidly gained in popularity through their increased accessibility to a wider bioimaging community and their versatility across biomedical imaging modalities (Bernabeu et al., 2020, Gómez-de-Mariscal et al., 2021) When successfully trained, ML approaches significantly reduce the work of generating segmentation, removing a potential bottle neck from an image-based modelling pipeline. For imaging techniques where large, open-source, ground truth data exist e.g. clinical imaging modalities, ML approaches have been particularly powerful (Willemink et al., 2020). Where ground truth data is lacking, e.g., for novel imaging modalities or disease application, ML methods, such as semi-supervised learning (Oliver et al., 2018) are being developed to mitigate the need for large ground truth datasets. Alternatively, citizen science approaches which aim to generate large ground truth data sets are expanding into the biomedical imaging space (Spiers et al., 2021).

Multimodal imaging approaches are increasingly employed which span biological scales of interest and have variable contrast (Cebulla et al., 2014, d’Esposito et al., 2018). Integrating these datasets provides complementary information but increases the complexity of image processing through the need to align these datasets.

A final challenge for image processing is the optimisation and validation of the output. Image processing pipelines tend to have high numbers of parameters that are challenging to estimate. One emerging strategy is to use mathematical techniques such as inverse problem solving and modelling to refine parameter choices and extract mechanistic information from the underpinning datasets (Cebulla et al., 2014).

## 4 Mathematical model development

Mathematical models rely on assumptions about the mechanisms underpinning a biological system’s behaviour. These are often formulated in terms of conservation laws (for example mass, momentum, energy), described through partial differential equation (PDE) systems which rely on the provision of model parameters and system behaviour at its boundaries. For example, the Navier-Stokes equations have been widely used to describe blood flow dynamics, require prescription of haemodynamic parameters such as blood density viscosity, and flow velocities or pressures at inlets or outlets.

An alternative strategy is to utilize ML, in which the model is learned from underlying patterns in the data without explicit programming of physical laws. This could significantly increase the speed of computation (Itu et al., 2016) compared to physical modelling in isolation. Increasing research effort is also being focused on ‘grey box’ modelling, in which ML (‘a black-box’ approach) is combined with mechanistic mathematical models (‘white box’) so that the underlying biophysics informs the learning (Deist et al., 2019).

The first challenge is to identify which mechanisms are required to build a representative model. Imaging can provide measures of blood flow at a range of length scales: organ/tissue scale for MRI, single vessel for confocal) (Daly and Leahy, 2013). There remain numerous open challenges including the scale of measurement (e.g., measuring blood flow in the microcirculation in situ), how to infer information on the mechanisms at play from the imaging data, and how to define the modelling domain.

Once the modelling domain is defined from an imaging data set, a set of constraints that define the model behaviour must be provided at the boundaries. These constraints are termed boundary conditions and together with the PDE system, form the Boundary Value Problem (BVP), i.e. the complete model to be solved. In most cases these conditions are complex to establish, in part because the boundaries of a given physical system can be arbitrary and because of a paucity of physical measurements at these boundaries. This is particularly true for complex biological systems which are characterised by multiple interacting features spanning length and time scales.

Developing integrated imaging and modelling frameworks provides a promising opportunity to address these challenges and there are numerous literature examples. (Pirola et al., 2017) developed a patient-specific model of aortic flow benchmarked against velocity profile data at the inlet of the penetrating aorta. However, such approaches are limited when the boundary data are difficult to acquire, for instance when predicting the link between structure and function in the microcirculation. Here, the imaging challenge remains to resolve the microcirculatory structures in situ whilst also acquiring functional measures such as pressures and flows. Mathematical models have been developed to interpolate between available structural and functional data where concurrent measurements are lacking. For example, (Cruz Hernández et al., 2019) described blood flow in the cortical microvasculature using a representative periodic boundary condition to accommodate for uncertainty in boundary pressures and flows. Schmid at al. (Schmid et al., 2017) embedded a microcirculatory network of interest in a much larger synthetic capillary network for which boundary data were less sensitive to impose. In both examples, the approach introduces biases into the model prediction, but provides a tool for bridging the gaps in measured fine-scale structural and functional data.

**Fig. 2:**
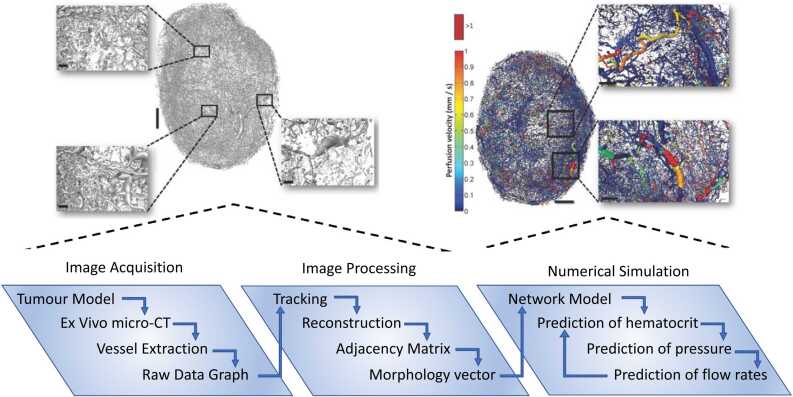
The Imaging-Modelling pipeline applied to tumours. Image acquisition, segmentation and numerical simulation used by (Stamatelos et al., 2014) to predict blood flow at the scale of a whole tumour whilst keeping the details of the microarchitecture. Here we include data from Fig. 1, 6 in (Stamatelos et al., 2014), with permission from the publisher.

It remains rare to have access to complete structural and functional datasets for whole organs, motivating the need for models that correlate data across scales whilst remaining computationally tractable. (d’Esposito et al., 2018) reconstructed the vasculature of whole tumours in 3D from *ex-vivo* OPT images. The authors developed an iterative algorithm to match flow predictions of their to measured, courser scale perfusion data from MRI through the choice of boundary conditions. The authors extended their approach to explore the relationship between model parameters and predictions through extensive sensitivity analyses (Sweeney et al., 2019). Another example applied to tumour physiology is summarised in Fig. 2 (Stamatelos et al., 2014). Such approaches are essential to understand the role of model parameters and provide mechanistic insights. Integrated, deterministic approaches such as these present exciting opportunities, but require advanced, multimodal imaging combined with computationally-efficient simulation and sufficient computing power to handle the volume of data.

In addition, dedicated experiments can be performed to isolate and quantify the role of individual mechanisms. For example, haemodynamic modelling characterises the link between blood flow and pressure via the blood viscosity, including the complex interplay between red blood cells, surrounding fluids and network structures. Pries and co-workers combined experiments in glass tubes (Pries et al., 1992), in situ imaging (Pries et al., 1989) and modelling (Pries et al., 1990) to define these relationships, which have been widely taken up by the microcirculation community. However, multidisciplinary studies which develop modelling and experimental/ imaging studies hand-in-hand remain seldom, presenting a significant obstacle to model validation.

## 5 Cross-validation and prediction

The goal when modelling biological processes is to better understand and predict the behaviour of systems which are challenging to decipher using experiments in isolation. In turn, imaging and experiments must inform model development (Fig. 1). Model validation, or the ability of a model to reproduce existing data, is critical and requires matching imaging data and model predictions whilst mitigating for the limitations of both. For instance, MRI generates large yet coarse tissue images and therefore are best paired to tissue-scale models which average (rather than explicitly model) the tissue microenvironment. This is demonstrated by (Slator et al., 2018) who compare clinical MRI data on placenta perfusion against different multicompartment models that link water diffusion to tissue microarchitecture. By comparison, (Gagnon et al., 2016) reconstructed a small section of the murine cortical vasculature using multi-photon microscopy and measured blood flow and oxygen transport at the scale of a single capillary, requiring a corresponding model at the same scale to enable validation.

Beyond model formulation, imaging processing algorithms themselves require validation. Segmentation algorithm predictions, for example, for a range of image processing pipelines, have been compared to *gold standards* acquired either manually or from synthetic data (Bernabeu et al., 2020, d’Esposito et al., 2018). Whilst manual segmentations are often regarded as the benchmark and used to inform modelling, they are highly labour intensive and not easily reproducible. This has motivated the development of machine learning approaches and counterpart training datasets (Bernabeu et al., 2020) to streamline reliability.

## 6 Conclusion

The combination of cutting-edge, integrated image modalities, image processing and mathematical modelling has the potential to unlock information on the behaviour of biological systems at an unprecedented level. However, significant bottlenecks remain, such as in-situ imaging through time, processing and simulation times and validation against ground truth data. Multidisciplinary approaches involving the concurrent development of mathematical models and experimental/imaging modalities remain rare but provide significant opportunity. Such developments will be accelerated through open-source sharing of complete and annotated data sets and models, to promote cross-community collaboration and stimulate the development of novel techniques (Regev et al., 2017, Snyder et al., 2019).

## Competing interests

The authors have no competing interests to declare.
